# Diagnostic Significance of Ki-67, p53, and Mucin Glycoprotein Expression in Colorectal Serrated Lesions: Further Immunohistochemical Studies

**DOI:** 10.3390/diagnostics16132099

**Published:** 2026-07-04

**Authors:** Ivan Ilić, Pavle Ranđelović, Ilija Golubović, Milan Lazarević, Maja Jovičić Milentijević, Biljana Radovanović Dinić, Ivana Đorđević, Jana Cvetković, Milica Veljković

**Affiliations:** 1 Center for Pathology and Pathological Anatomy, University Clinical Center Niš, Faculty of Medicine, University of Niš, 18000 Niš, Serbia; ilicko81@gmail.com (I.I.); majajmi@gmail.com (M.J.M.); ivanadjordjevic01.12@gmail.com (I.Đ.); 2 Department of Physiology, Faculty of Medicine, University of Niš, 18000 Niš, Serbia; milica.veljkovic@gmail.com; 3 Clinic for Digestive Surgery, University Clinical Center Niš, 18000 Niš, Serbia; golubovicilija@yahoo.com; 4 Department of Immunology, University Clinical Center Niš, Faculty of Medicine, University of Niš, 18000 Niš, Serbia; dr_m.lazarevic@hotmail.com; 5 Clinic for Gastroenterology and Hepatology, University Clinical Center Niš, Faculty of Medicine, University of Niš, 18000 Niš, Serbia; bikius@yahoo.com; 6 Department for Pathology, General Hospital Leskovac, 16000 Leskovac, Serbia; jana.cvetkovic92@gmail.com

**Keywords:** serrated lesions, mucin glycoproteins, colorectal adenoma, hyperplastic polyp, Ki-67, p53

## Abstract

**Background/Objectives:** Colorectal serrated lesions are highly heterogeneous at the micromorphological level. Their morphology can range from seemingly harmless, as in the case of hyperplastic polyps with little to no neoplastic potential, to lesions that can undergo malignant transformation within a short period of time. The serrated morphology of these lesions is primarily a consequence of morphological changes resulting from the enlargement and budding of the adenoma, as well as the migration of its epithelium at the crypt level. This creates elevations that resemble saw teeth. In this study, the serrated morphology was highlighted immunohistochemically using the MUC2 marker. **Results:** MUC5AC immunohistochemical expression exhibited greater variability in positivity and intensity compared to MUC2 expression. Pancryptic MUC5AC positivity was present in 75% of TSA cases and in 75% of malignantly transformed serrated lesions. This correlates with advanced histologic lesions and dysplasia development within the serrated pathway of carcinogenesis. MUC5AC is a useful marker for detecting polyps undergoing malignant transformation but not for distinguishing between sessile serrated lesions and hyperplastic polyps. Analysis of Ki-67 proliferative activity in serrated lesions in this study showed that a shift in the proliferative zone along the entire crypt was statistically significant only in TSA (*p* < 0.01). p53 expression increases from low-grade to high-grade dysplasia and malignant transformation of serrated lesions. This study confirmed that p53 expression in the upper half of the crypt was present in 11% of sessile serrated lesions without dysplasia and in 18% with dysplasia. In malignantly transformed serrated lesions, pancryptic positivity was present in 25% of cases. **Conclusions:** A marker panel consisting of mucin glycoproteins, Ki-67, and p53 could be useful in assessing the risk level of malignant transformation in serrated lesions.

## 1. Introduction

The 2019 WHO classification nosologically divides serrated lesions into hyperplastic polyps, sessile serrated lesions with and without dysplasia, and traditional serrated adenomas. We reported on the morphology and differential diagnostic expression of intermediate filament proteins of these lesions in a previous publication [1,2]. Some serrated lesions, such as sessile serrated lesions, are difficult to diagnose and distinguish from other types of colorectal polyps due to their overlapping histological characteristics [3]. Most serrated lesions are asymptomatic and are incidental findings during endoscopy. Since bleeding from serrated polyps is rare and most serrated polyps are sessile, stool blood testing and virtual colonoscopy are ineffective screening methods [4].

Sessile serrated lesions attract special attention due to their potential to transform malignantly into colorectal carcinoma through the serrated pathway [3], and the risk of malignant transformation varies greatly [5]. The role of mucin glycoproteins in the serrated pathway of carcinogenesis has not been fully elucidated, nor have differences in mucin expression between lesions of the right and left colon been observed [3,6].

The main mucin secreted by intestinal goblet cells is Mucin 2 (MUC2). Under physiological conditions in the colon, MUC2 forms two layers of mucus that protect the epithelial surface and support epithelial cell adhesion and signal transduction [7]. Mucin 5AC (MUC5AC) expression has been observed in the colon during chronic inflammation, infection, adenoma, and carcinoma [8]. However, pathologists have noted abnormal expression patterns in colorectal polyps and carcinomas. The expression pattern itself can serve as a criterion for detecting sessile serrated lesions [8,9].

MUC5AC is a marker with varying expression in visually similar serrated lesions with different biological potentials, such as hyperplastic polyps and sessile serrated lesions. Therefore, it can be used to differentiate between them [10]. Differences in MUC5AC expression between sessile serrated lesions and other polyp subtypes are consistent with previous research on this topic. Based on literature from immunohistochemical studies, MUC5AC expression is consistently upregulated in sessile serrated lesions [8]. MUC5AC expression is considered a potential marker for assessing the risk of malignant transformation because hypomethylation of the MUC5AC gene occurs early in the serrated pathway of carcinogenesis in microvesicular hyperplastic polyps and sessile serrated lesions [11].

Although there are no immunohistochemical markers specific to Traditional Serrated Adenoma (TSA), it has been shown that MUC5AC expression is more prevalent in the recently described category of mucin-rich TSAs than in traditional TSAs [12].

Recent semiquantitative analyses revealed a significant increase in MUC2 and MUC5AC immunoreactivity in sessile serrated lesions [13], such that all sessile serrated lesions express MUC2 and MUC5AC, while decreased expression is usually seen in areas with high-grade dysplasia [3]. Some studies have shown that reduced MUC2 expression is present in colorectal carcinoma. Cases of colorectal carcinoma with positive MUC2 immunohistochemical expression have a better prognosis and the lowest metastatic potential [14].

Increased proliferative activity and enhanced cell renewal in serrated lesions result from abnormal stem cell behavior in different proliferative compartments along the crypt. This disorganized proliferative activity explains why goblet cells are now present in the bases of the crypts. The Ki-67 proliferative marker helps assess the shifted proliferative zone [15]. Typically, Ki-67 proliferative activity is localized in the lower third of crypts and exhibits asymmetric distribution in sessile serrated lesions. It is particularly intense in lesions with dysplasia [3]. Dysplasia is present in some sessile serrated lesions. The boundary between dysplastic and non-dysplastic areas is usually clearly defined. This helps pathologists identify the extent of dysplasia and assess the risk of malignant transformation. However, due to the pronounced micromorphological heterogeneity of these lesions, grading dysplasia is often subjective and therefore not recommended by the WHO [1]. Sessile serrated lesions usually exhibit more than one pattern of dysplasia [16].

One of the terminal events in the malignant transformation of conventional adenomas is the TP53 mutation [17], and certain studies have highlighted the rare occurrence of these mutations in sessile serrated lesions [18]. Some authors have noted the absence of TP53 expression in areas of low-grade dysplasia in these lesions, suggesting that this event occurs earlier in the malignant transformation process than in conventional adenomas [3]. The reported frequency of TP53 mutations ranges from 33–45% in hyperplastic polyps, 43.9% in sessile serrated lesions, and 40–61.5% in TSAs [19,20].

Increased epithelial proliferation and alterations in cell control proteins are possible mechanisms of colorectal oncogenesis. The discovery of antibodies for proliferative antigens allows for the quick and easy determination of the proliferative status of adenomas, and they can be used routinely on paraffin-embedded material. The intense immunoreaction of these markers in adenomas with high-grade dysplasia suggests a strong link between these adenomas and colorectal carcinoma. Ki-67 and p53 immunohistochemistry have received the most attention, especially since they are routinely performed in diagnostic laboratories [21].

Unlike previous studies that mainly evaluated overall marker positivity, the present study focuses on the distribution of immunohistochemical expression within different crypt compartments and evaluates the combined diagnostic utility of MUC2, MUC5AC, Ki-67, and p53 across the full spectrum of serrated colorectal lesions. Furthermore, in comparison to our previous research, this study expands the immunohistochemical marker panel, providing a comprehensive set of diagnostic tools that can, in challenging cases, significantly aid in the precise differential evaluation of serrated lesion subtypes.

## 2. Materials and Methods

### 2.1. Study Design and Sample Selection

This retrospective study was based on tissue material collected over a four-year period and referred to the Gastrointestinal Pathology Unit for histopathological review or a ‘second-look opinion’ from other laboratories and regional pathology centers. No consecutively received cases were excluded from the study due to technical or diagnostic difficulties, such as poor specimen orientation within the paraffin block, suboptimal staining, or insufficient tissue volume; notably, this inclusive approach resulted in a diagnosis of unclassified serrated lesion in two cases. When the reviewed biopsy material comprised two or more paraffin blocks from the same lesion, the selection of the most representative specimen for subsequent immunohistochemical analysis was prioritized based on the following hierarchical criteria: First, areas demonstrating a higher grade of dysplasia (if present); second, the presence of the most characteristic architectural alterations defining a specific serrated lesion (e.g., serration confined to the upper or lower half of the crypts, crypt distortion with asymmetric proliferation, or aberrant crypt foci); and finally, the overall volume of tumor tissue within the block.

This study was approved by the Institutional Ethics Committee of the University Clinical Center Niš (Approval No. 19074/3 on 11 February 2026). Histopathological evaluation of all slides was performed independently by two pathologists. Any divergent opinions regarding the diagnoses or immunohistochemical scoring were discussed until a consensus was reached.

### 2.2. Patient Cohort and Tissue Sourcing

Similar to our previous study, we performed a panel analysis of immunohistochemical markers to evaluate mucin expression, the Ki-67 proliferative index, and p53 immunohistochemical expression pattern in colorectal serrated lesions. The studied cohort (*n* = 109) comprised 34 hyperplastic polyps (HPs), 27 sessile serrated lesions without dysplasia, 11 sessile serrated lesions with dysplasia, 6 TSAs, 21 mixed serrated lesions, 8 malignantly transformed serrated lesions, and 2 unclassified serrated lesions, primarily classified according to the World Health Organization (WHO) recommendations. Two pathologists diagnosed and histopathologically confirmed all cases. For this study, we used tissue samples of serrated lesions obtained as colonoscopic biopsies or polypectomy specimens from the Clinic for Gastroenterology and Hepatology at the University Clinical Center Niš. The samples were also obtained as part of partial and complete colectomies in patients who were surgically treated at the University Clinical Center Niš. Well-oriented sections that adequately visualize the crypt bases were crucial for precisely assessing lesion architecture. All samples had been processed and archived in paraffin blocks together with pathohistological and clinical documentation at the Center for Pathology and Pathological Anatomy of the University Clinical Center in Niš.

### 2.3. Immunohistochemical Staining Protocol

For the purposes of immunohistochemical staining, the microscopically most characteristic sample (region) was taken from the lesion area and subjected to this specific procedure in the form of a single paraffin block. Based on the primary morphological assessment performed on HE sections, one block was selected containing either the entire tissue sample of the serrated lesion or a representative region from larger-diameter serrated lesions. Sections approximately 4 µm thick were cut from the paraffin blocks using a microtome, adhered to “super frost” slides, and stained immunohistochemically for MUC5AC (Monoclonal Mouse Anti-Human MUC5AC Antigen; Ready-to-use; Clone CLH2; Code IR661), MUC2 (Monoclonal Mouse Anti-Human MUC2 Antigen; Ready-to-use; Clone CCP58; Code IR658), p53 (Monoclonal Mouse Anti-Human p53 Protein; Ready-to-use; Clone DO-7; Code IR616), and Ki-67 (Monoclonal Mouse Anti-Human Ki-67 Antigen, Ready-to-use; Clone MIB-1; Code IR626).

After deparaffinization and hydration of the sections through xylene and a series of alcohols in decreasing concentrations, antigen retrieval was performed in a microwave oven for 20 min in citrate buffer, followed by cooling at room temperature, rinsing, and blocking of endogenous peroxidase with 3% hydrogen peroxide. The sections were rinsed in PBS (Phosphate-Buffered Saline) buffer pH = 7.4, after which the primary antibody was applied with an incubation period of 40 min at room temperature. Following the PBS rinse, the Labelled Streptavidin-Biotin 2 System, Horseradish Peroxidase (LSAB2 System-HRP, 15 mL, Code K0673, DAKO Cytomation, Copenhagen, Denmark) was applied, containing yellow and red LINK and incubated for 20 min each, with PBS rinsing between each step. All antibodies used were from the same manufacturer (DAKO, Glostrup, Denmark), and the visualization system used was En Vision with DAB chromogen (DAKO Cytomation, Copenhagen, Denmark). Background staining was performed using Mayer’s hematoxylin (Merck, Darmstadt, Germany) with standard exposure time and other standardized parts of the procedure; finally, the sections were dehydrated and cleared (briefly in 75% ethanol, briefly in 96% ethanol, 5 min in absolute alcohol, and 10 min in xylene) and coverslipped using DPX. To ensure the validity of the immunohistochemical staining, appropriate positive and negative controls were included in each run. Positive expression for MUC2 and MUC5AC was identified as brown cytoplasmic staining, with gastric mucosa as a positive control for MUC5AC and adjacent normal colonic mucosa as an internal positive control for MUC2. For the nuclear markers Ki-67 and p53, positive expression was defined as brown nuclear staining, with external tissue sections of the appendix or tonsil as positive controls. Negative controls were established by omitting the primary antibody on control sections. Moreover, most samples inherently contained internal negative and positive controls. For instance, the transitional colonic mucosa adjacent to the sessile serrated lesion, as well as the normal colonic mucosa, served as internal negative controls for MUC5AC expression, demonstrating a complete absence of specific brown immunoreactivity.

### 2.4. Histopathological Evaluation and Scoring Framework

The immunohistochemical markers MUC2, MUC5AC, Ki-67, and p53 were selected as a comprehensive panel to concurrently evaluate epithelial cell lineage differentiation, architectural proliferative tracking, and critical genomic mutation pathways driving the serrated neoplastic cascade. The results of immunohistochemical analyses were assessed semiquantitatively by evaluating the intensity of reactivity (graded as: 0, absent; 1+, weak; 2+, moderate; 3+, strong) and determining the percentage of positive tumor cells. A standard baseline threshold of 10% was utilized to define general positivity for MUC5AC, MUC2, and Ki-67, serving as a widely accepted cut-off in diagnostic surgical pathology to minimize non-specific background artifacts.

For p53, rather than evaluating simple positivity thresholds, the p53 immunohistochemical expression patterns were categorized into aberrant (mutant-like) or wild-type (normal) phenotypes based on well-established reproducible criteria [17], which serve as validated surrogates for underlying TP53 mutations. An “overexpression pattern” was defined as strong, diffuse nuclear expression in 80% or more of the tumor cells (irrespective of cytoplasmic staining). A “null-pattern” was defined as the complete absence of any nuclear p53 immunoreactivity within the tumor epithelium, evaluated against positive internal controls (adjacent stromal or inflammatory cells). Staining configurations that did not meet the criteria for either overexpression or complete loss were categorized as a “wild-type pattern” and were considered non-aberrant (negative) in our analysis.

To ensure high reproducibility and minimize subjectivity in assessing crypt architecture, crypt compartment scoring for the remaining markers was operationally defined based on the topographical distribution of staining along the longitudinal axis of well-oriented crypts: (1) “Lower-half” positivity was defined as staining strictly confined to the basal 50% of the crypt length; (2) “Upper-half” positivity was defined as staining restricted predominantly to the luminal/surface 50% of the crypts; (3) “Pan-cryptic” positivity was defined as diffuse staining involving more than 75% of the total crypt length. These architectural cut-offs were specifically established to objectively trace the upward displacement of the proliferative zone and aberrant maturation gradients that characterize serrated tumorigenesis. All tissue slides and immunohistochemical stains were evaluated independently by two experienced pathologists who were blinded to each other’s initial scores. Interobserver agreement was explicitly assessed; borderline or discordant cases were jointly reviewed using a multi-head microscope, and divergent opinions were discussed until a final diagnostic and scoring consensus was reached.

### 2.5. Statistical Analysis

Statistical processing of the data was performed using IBM SPSS Statistics software (version 25.0; IBM Corp., Armonk, NY, USA). Descriptive statistical methods were used to display the distribution of clinicopathological features, with numerical data explicitly presented as both absolute counts (frequencies) and percentages to prevent overinterpretation in smaller subgroups.

To examine the association between histological types and immunohistochemical expression patterns (MUC2, MUC5AC, Ki-67, and p53), Fisher’s Exact Test was utilized. Given that more than 20% of the cells in the contingency table had an expected count of less than 5, the Monte Carlo method (based on 10,000 sampled tables) was applied to ensure the precision of the *p*-values.

For trend analysis via the Linear-by-Linear Association test (applied to p53 and Ki-67), the histological categories were ordered as follows: Hyperplastic Polyps (HPs) → Sessile Serrated Lesions (SSLs) without dysplasia → SSLs with dysplasia → Mixed Serrated Lesions/Traditional Serrated Adenomas (TSAs) → Malignantly Transformed Lesions. This ordering does not assume a single, linear biological continuum; rather, it represents a progressive gradient of cytological atypia, architectural complexity, and cumulative neoplastic risk.

Diagnostic performance parameters (Sensitivity and Specificity) were calculated using 2 × 2 contingency tables alongside their respective 95% Confidence Intervals (CI) using the Wilson score method. Due to the exploratory nature of this study involving multiple markers, formal adjustments for multiplicity (e.g., Bonferroni correction) were not strictly applied to preserve statistical power within small subgroups; however, the resulting increased risk of Type I error is explicitly acknowledged as a study limitation.

A *p*-value of < 0.05 was considered statistically significant for all tests, while values of < 0.001 were interpreted as highly significant.

## 3. Results

### 3.1. MUC5AC Expression in Serrated Lesions

The analysis of MUC5AC distribution revealed a highly statistically significant difference in expression patterns across the examined histological subtypes (Fisher’s Exact Test = 62.838; *p* < 0.001).

Preserved marker expression was predominantly observed in benign lesions, such as hyperplastic polyps (HPs) and sessile serrated lesions (SSLs) without dysplasia. Specifically, upper-half crypt positivity was found in 61.8% of HPs (21/34) and 74.1% of SSLs without dysplasia (20/27).

**Figure 1:**
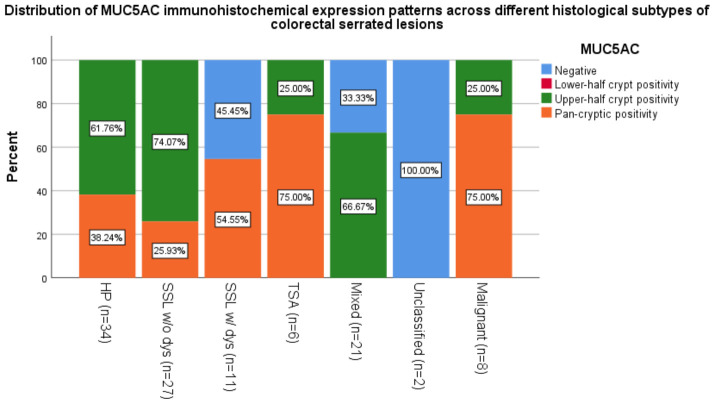
Distribution of MUC5AC immunohistochemical expression patterns across different histological subtypes of colorectal serrated lesions (n = 109). HP—Hyperplastic polyp; SSL w/o dys—Sessile serrated lesion without dysplasia; SSL w/dys—Sessile serrated lesion with dysplasia; TSA—Traditional serrated adenoma; Mixed—Mixed serrated lesion; Unclassified—Unclassified serrated lesion; Malignant—Malignantly altered serrated lesion.

In contrast, a complete loss of MUC5AC expression was exclusively associated with more advanced lesions, occurring in 45.5% (5/11) of SSLs with dysplasia and 33.3% (7/21) of mixed serrated lesions. Traditional serrated adenomas (TSAs) exhibited a distinct profile, with full-crypt (pan-cryptic) positivity present in 75% of cases. These findings suggest that the loss or altered distribution of MUC5AC expression correlates with histological progression and the development of dysplasia within the serrated pathway (Figure 1).

### 3.2. MUC2 Expression in Serrated Lesions

The immunohistochemical analysis of MUC2 expression revealed a highly uniform distribution pattern across the majority of the examined serrated lesions. Statistical analysis demonstrated a highly significant difference in the distribution of expression patterns (Fisher’s Exact Test = 24.614; *p* < 0.001). This significance was primarily driven by the complete absence of expression in unclassified lesions and specific variations within the hyperplastic polyp group.

A predominant pan-cryptic (full crypt) positivity was the hallmark of MUC2 expression in this cohort. This pattern was observed in 100% of cases for the following categories: sessile serrated lesions (SSLs) without dysplasia (27/27), SSLs with dysplasia (11/11), traditional serrated adenomas (6/6), mixed serrated lesions (21/21), and malignantly altered serrated lesions (8/8).

**Figure 2:**
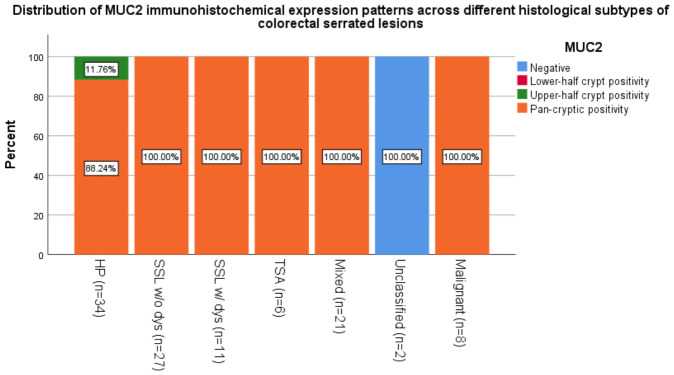
Distribution of MUC2 immunohistochemical expression patterns across different histological subtypes of colorectal serrated lesions (n = 109). HP—Hyperplastic polyp; SSL w/o dys—Sessile serrated lesion without dysplasia; SSL w/dys—Sessile serrated lesion with dysplasia; TSA—Traditional serrated adenoma; Mixed—Mixed serrated lesion; Unclassified—Unclassified serrated lesion; Malignant—Malignantly altered serrated lesion.

Hyperplastic polyps (HPs) showed slight variability; while the vast majority exhibited pan-cryptic expression (88.2%; 30/34), a small subset (11.8%; 4/34) demonstrated positivity restricted to the upper half of the crypts. Complete loss of MUC2 expression was noted only in the unclassified serrated lesion group (2/2). In contrast to other markers, the Linear-by-Linear Association was not significant (*p* = 0.243), confirming that MUC2 acts as a stable marker of the serrated pathway, maintaining consistent pan-cryptic expression regardless of the degree of histological progression or the presence of dysplasia (Figure 2).

### 3.3. Ki-67 Proliferative Activity in Serrated Lesions

The analysis of Ki-67 immunohistochemical expression revealed a highly statistically significant difference in the distribution of the proliferative zone across the examined histological subtypes (Fisher’s Exact Test = 70.778; *p* < 0.001). Given that 82.1% of the cells had an expected count of less than 5, the significance was confirmed using the Monte Carlo method, which also yielded maximum significance (*p* < 0.001).

In benign lesions, proliferative activity remained strictly localized to the basal segments. In hyperplastic polyps (HPs), proliferation was restricted to the lower half of the crypt in 94.1% of cases (32/34). A similar pattern was observed in all sessile serrated lesions (SSLs) without dysplasia (100%, 27/27), where the proliferative zone was confined to the basal half.

In sharp contrast, traditional serrated adenomas (TSAs) exhibited a completely different profile, with 100% of cases (6/6) showing pan-cryptic (full crypt) positivity. This indicates a complete expansion of the proliferative zone, serving as a clear differential diagnostic parameter compared to HPs and SSLs without dysplasia.

**Figure 3:**
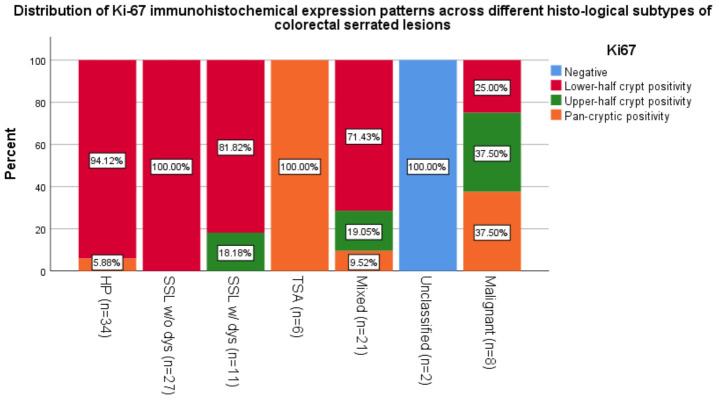
Distribution of Ki-67 immunohistochemical expression patterns across different histological subtypes of colorectal serrated lesions (n = 109). HP—Hyperplastic polyp; SSL w/o dys—Sessile serrated lesion without dysplasia; SSL w/dys—Sessile serrated lesion with dysplasia; TSA—Traditional serrated adenoma; Mixed—Mixed serrated lesion; Unclassified—Unclassified serrated lesion; Malignant—Malignantly altered serrated lesion.

In more complex and malignantly altered lesions, a significant shift in proliferation toward the surface was observed. Among malignantly altered serrated lesions, only 25% (2/8) retained basal proliferation, while the remainder showed expansion into the upper segments or the full crypt. The Linear-by-Linear Association test (Value = 14.221, *p* < 0.001) confirms a significant trend: As lesions progress toward more advanced histological stages, the proliferative zone progressively expands along the entire length of the crypt (Figure 3).

### 3.4. p53 Expression in Serrated Lesions

The immunohistochemical analysis of p53 expression revealed a highly statistically significant difference in staining patterns across the examined histological subtypes (Fisher’s Exact Test = 39.398; *p* < 0.001). Due to the nature of the data distribution and the low expected frequencies in several categories (75.0% of cells with an expected count < 5), significance was confirmed using the Monte Carlo method (*p* < 0.001).

The majority of the examined lesions exhibited negative p53 expression (55.0%; 60/109). In more benign forms, such as hyperplastic polyps (HPs) and sessile serrated lesions (SSLs) without dysplasia, expression was either entirely absent or restricted to the lower half of the crypts, which is consistent with a “wild-type” protein distribution. No cases of pan-cryptic (full crypt) positivity were observed in these specific groups.

**Figure 4:**
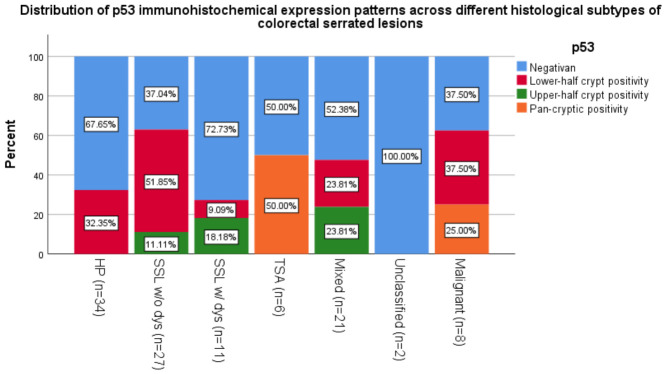
Distribution of p53 immunohistochemical expression patterns across different histological subtypes of colorectal serrated lesions (n = 109). HP—Hyperplastic polyp; SSL w/o dys—Sessile serrated lesion without dysplasia; SSL w/dys—Sessile serrated lesion with dysplasia; TSA—Traditional serrated adenoma; Mixed—Mixed serrated lesion; Unclassified—Unclassified serrated lesion; Malignant—Malignantly altered serrated lesion.

In contrast, aberrant expression patterns were noted in advanced lesions. Traditional serrated adenomas (TSAs) showed a distinct profile, with 50% of cases (3/6) exhibiting pan-cryptic positivity. Similarly, 25% (2/8) of malignantly altered serrated lesions demonstrated expansion of the marker throughout the entire crypt depth. The Linear-by-Linear Association test (Value = 5.391, *p* = 0.020) suggests a significant trend: As the histological severity of the lesion increases, there is a higher probability of expanded and aberrant p53 expression patterns, potentially indicating genetic instability associated with malignant transformation (Figure 4).

The diagnostic performance parameters summarized in Table 1 evaluate the utility of specific immunohistochemical patterns strictly for within-serrated-lesion differentiation and risk stratification. The diagnostic target for each marker was selected based on observed biological shifts during progression.

Specifically, pan-cryptic nuclear Ki-67 expression demonstrated a sensitivity of 100% (95% CI: 61.0–100.0%) for identifying the Traditional Serrated Adenoma (TSA) phenotype against all other serrated subtypes within this cohort. For identifying advanced histological features (defined as the presence of dysplasia or malignancy within the serrated pathway), a complete loss of MUC5AC expression exhibited a specificity of 100% (95% CI: 94.3–100.0%) and a sensitivity of 39.1% (95% CI: 26.5–53.5%). Similarly, aberrant pan-cryptic p53 accumulation served as a highly specific (100%, 95% CI: 96.1–100.0%) but less sensitive (35.7%, 95% CI: 16.3–61.2%) indicator for advanced TSA or malignant transformation.

While markers such as p53 pan-cryptic positivity and MUC5AC loss of expression showed moderate sensitivity, their absolute (100%) specificity highlights their clinical value. This indicates that while these molecular alterations do not manifest in every advanced case, their presence provides a definitive, highly specific diagnostic indicator within the studied groups for high-risk progression and malignant transformation.

Lastly, using a standard 10% positivity cut-off, pan-cryptic MUC2 expression served as a highly sensitive lineage-specific indicator (94.5%, 95% CI: 88.5–97.5%), confirming the stable secretory identity across the serrated spectrum, though intra-lineage specificity could not be calculated (N/A) due to the absence of a non-serrated control cohort.

## 4. Discussion

According to Longacre and Fenoglio-Preiser, serrated adenomas are distinguished from other colorectal neoplasms by their saw-toothed epithelial surface, prominent mitoses in the upper crypts, prominent nucleoli, absence of a thickened collagen layer, and a nucleocytoplasmic ratio that places them between hyperplastic polyps and conventional adenomas. This morphology is primarily a consequence of the adenoma’s enlargement and budding, as well as the epithelium’s migration at the crypt level, creating elevations that resemble saw teeth [22]. In this study, serrated morphology was highlighted using the MUC2 marker. In our previous research, serrated morphology was highlighted using CK7 and CK20 in hyperplastic polyps (*p* < 0.01).

While histopathological differences between serrated lesion subtypes are usually sufficient for pathologists to make a definitive diagnosis, differences in the expression of mucin glycoproteins and cytokeratins remain a subject of discussion [23,24]. In this study, serrated lesions with dysplasia (SSL) and mixed serrated lesions were statistically significantly more often negative for MUC5AC mucin glycoprotein than other serrated lesions (*p* < 0.01). Loss of MUC2 mucin glycoprotein expression was a statistically significant differential diagnostic feature of unclassified serrated lesions compared to all other serrated lesions (*p* < 0.01). These results demonstrate that the mucin glycoprotein and cytokeratin panel analyzed in our previous study can be used when morphological characteristics are insufficient for diagnosing serrated lesions. In accordance with the WHO’s most recent position on the classification of serrated lesions [1] and the results of this analysis, a diagnosis of an unclassified serrated lesion can be established if the applied marker panel is negative.

The isolated application of these immunohistochemical markers in routine histopathological practice may not be of paramount importance for distinguishing serrated lineage lesions from conventional tubular or villous adenomas, as the macro-differentiation between these two major pathways is generally straightforward. However, overlapping or subtle micromorphological features between specific serrated subtypes, compounded by suboptimal, small, or fragmented biopsy specimens, frequently generate challenging differential diagnostic dilemmas. In such borderline or ambiguous scenarios, the clinical utility of the evaluated marker panel becomes evident, serving as an effective ancillary diagnostic tool to guide precise subtyping and risk stratification strictly within the serrated spectrum.

**Figure 5:**
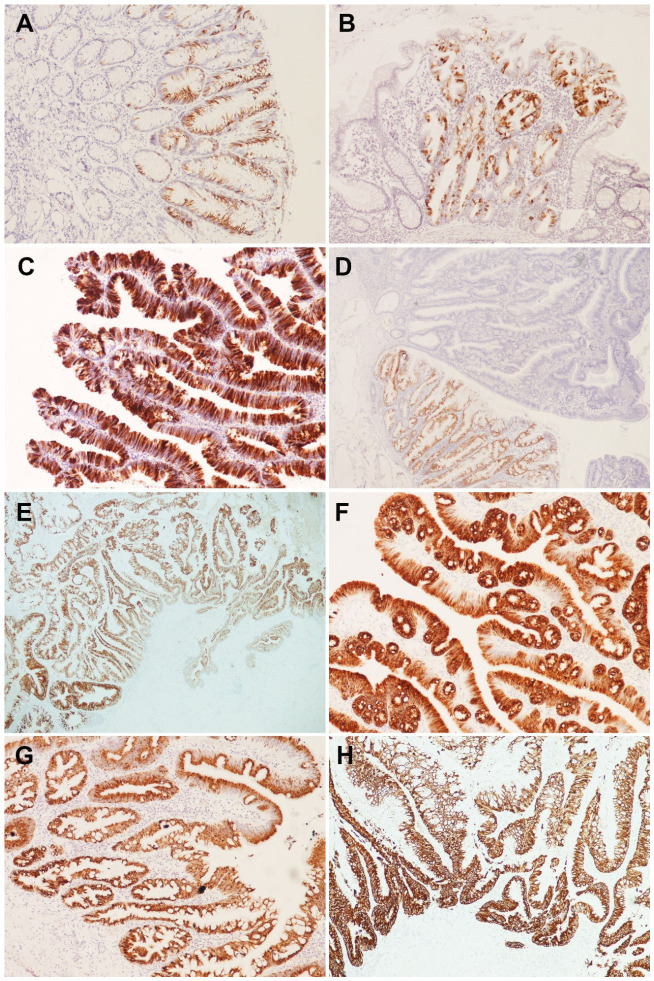
(**A**) MUC5AC immunohistochemical expression in the upper half of the crypts (right) in a hyperplastic polyp (LSAB2 × 100); (**B**) Sessile serrated lesion without dysplasia showing MUC5AC expression along the entire length of the crypts (LSAB2 × 100). Note the internal negative control of MUC5AC expression within the transitional colonic mucosa adjacent to the SSL (left third of the image) and within the normal colonic mucosa (lower right corner of the image), characterized by the complete lack of brown chromogen deposition; (**C**) Surface of a TSA with strong immunohistochemical expression of the mucin glycoprotein MUC5AC (LSAB2 × 100); (**D**) Mixed serrated lesion with positive MUC5AC immunoreactivity in the SSL component and negative MUC5AC expression in the TSA component (LSAB2 × 40); (**E**) Moderate to strong complete MUC5AC expression in a sessile serrated lesion of the appendix with dysplasia and malignant transformation (LSAB2 × 40); (**F**) Strong immunohistochemical expression of MUC2 glycoprotein in a TSA (LSAB2 × 100); (**G**) Mixed serrated lesion with MUC2 immunohistochemical positivity in both components (TSA + SSL) (LSAB2 × 100); (**H**) Complete and strong MUC2 immunohistochemical expression in a sessile serrated lesion of the appendix with dysplasia and malignant transformation (LSAB2 × 100).

Although this study did not specifically consider the mucin-rich TSA subtype, which has been shown to have MUC5AC expression significantly more frequently [12], the mere presence of pancryptic MUC5AC positivity in 75% of all TSA cases (Figure 5C), as well as in 75% of cases of malignantly transformed serrated lesions (Figure 5E), correlates with histologically advanced lesions and the development of dysplasia within the serrated pathway of carcinogenesis. This finding is consistent with the findings of other authors that MUC5AC is a useful marker for the detection of polyps undergoing the process of malignant transformation, but not for differentiating between sessile serrated lesions and hyperplastic polyps [25].

Findings from some studies indicate that MUC5AC expression is intense along the entire length of the crypt in sessile serrated lesions, whereas in hyperplastic polyps and TSAs, it is only focal [26]. In our study, it was shown to be present along the entire crypt in 26% of sessile serrated lesions without dysplasia and 55% of sessile serrated lesions with dysplasia. Furthermore, if we consider expression only in the upper half of the crypts as the equivalent of focal positivity, then it is present in 62% of hyperplastic polyps (Figure 5A), and in only 25% of TSAs, which again indicates successive changes during the histological progression of serrated lesions and an increase in the grade of dysplasia. This study confirmed the observations by certain authors that MUC5AC expression is not specific to sessile serrated lesions and that further research into the clinical use of MUC5AC could improve the detection of these lesions [8]. Although recent studies have semiquantitatively verified a significant increase in MUC2 and MUC5AC expression in sessile serrated lesions [13], they have not clearly determined the distribution of mucin glycoprotein expression in relation to the specific part of the crypt.

The grading of dysplasia in serrated lesions is not recommended by some authors [27], as dysplastic epithelium shows reduced mucin secretion proportional to the grade of dysplasia. The results of this study confirmed these claims in SSLs with dysplasia and mixed serrated lesions, which were statistically significantly more likely to lack the expression of the MUC5AC mucin glycoprotein (*p* < 0.01), but this was not the case for MUC2 mucin glycoprotein expression. Aberrant expression of MUC2 and MUC5AC mucin glycoproteins in a publication featuring multivariate regression analysis clearly showed differences between advanced lesions and hyperplastic polyps [28], which was also demonstrated in our study (Figure 5F,H), while other studies have highlighted that a low level of MUC2 mucin glycoprotein expression correlates with advanced precursor lesions and lesions with malignant transformation [29].

Similar to the observations by certain authors that MUC2 expression is constantly present in all serrated lesions [30], this study also showed that all serrated lesions, except for unclassified serrated lesions, exhibit excessive MUC2 positivity, at least in the upper half of the crypts of hyperplastic polyps. Such observations are not surprising, as the normal colonic mucosa also expresses MUC2, in contrast to non-mucinous adenocarcinomas [31]; therefore, the loss of MUC2 expression in this study can serve as a criterion for the detection of unclassified serrated lesions. On the other hand, other studies have shown that MUC2 expression patterns are important for the detection of sessile serrated lesions [8] or for differentiating malignantly transformed serrated lesions from non-mucinous adenocarcinomas [31]. Also, a multivariate analysis showed that the loss of MUC2 expression is significantly more pronounced in adenomas and adenocarcinomas compared to hyperplastic polyps [28], which in this study proved to be a specific feature only of unclassified serrated lesions, but not of hyperplastic polyps, which in a smaller percentage (11.8%) showed preserved MUC2 expression in the upper halves of the crypts.

A hallmark feature that distinguishes SSLs is the global and comprehensive distortion of normal crypt architecture, which is a direct consequence of alterations and shifts within the proliferative zone [15,32]. In these lesions, proliferative zones are no longer localized strictly at the crypt bases, but are instead displaced asymmetrically to one side of the crypt or the other. Ki-67 immunohistochemical staining highlights this abnormal, asymmetrical proliferation and reveals cells with goblet or gastric-foveolar differentiation at the very bases of the crypts [1]. In our research, there was no statistically significant shift of the proliferative zone in SSLs without dysplasia (*p* = 0.0003), while in SSLs with dysplasia, a shift of the proliferative zone toward the crypt surface was recorded in 18.2% of cases, though without statistical significance (*p* > 0.01).

**Figure 6:**
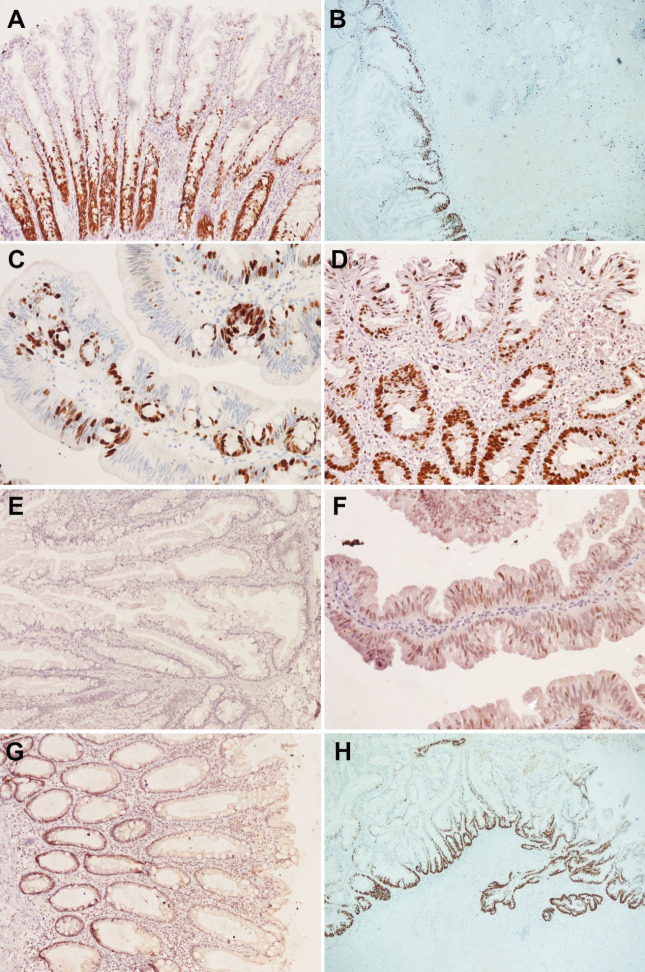
(**A**) Ki-67 proliferative index limited to the lower half of the crypts in a hyperplastic polyp (LSAB2 × 100); (**B**) Ki-67 immunoreactivity in the basal third of dilated crypts in a sessile serrated lesion with dysplasia (LSAB2 × 40); (**C**) Surface of a TSA where the Ki-67 proliferative index clearly demarcates ectopic crypt foci from the surrounding epithelium of villous projections (LSAB2 × 200); (**D**) Shift of the Ki-67 proliferative zone from the basal parts to the entire crypts in a hyperplastic polyp (LSAB2 × 200); (**E**) Focal basal p53 protein expression in an SSL without dysplasia (LSAB2 × 100); (**F**) Moderate to strong nuclear immunohistochemical p53 expression in a TSA (LSAB2 × 200); (**G**) Moderate to strong p53 immunoreactivity in the nuclei of epithelial cells in the lower halves of the crypts in a hyperplastic polyp (LSAB2 × 100); (**H**) Strong p53 immunohistochemical expression in the nuclei of tumor cells in a serrated adenocarcinoma arising from a sessile serrated lesion, in which p53 immunoreactivity is limited to the lower third of the crypts (LSAB2 × 40).

Conversely, although some literature indicates that mitoses are very rare in TSA and that there is no significant increase in the Ki-67 index compared to conventional adenomas [15], our findings do not support this. In this study, TSAs exhibited a statistically significant shift in the proliferative zone (demonstrating negativity in the lower half of the crypts; *p* < 0.01) as well as diffuse positivity across the entire length of the crypts (*p* < 0.01) compared to other serrated lesions, clearly indicating an elevated Ki-67 proliferative index. Furthermore, while certain studies suggest that cytological characteristics alone do not provide specific features for TSA beyond the presence of “ectopic” crypts that have lost their connection with the underlying muscularis mucosae [15], these ectopic formations, when inconspicuous on standard HE sections, can be clearly highlighted using the Ki-67 proliferative marker. This was confirmed in our research (Figure 6C), thereby underscoring the diagnostic value of Ki-67 in addition to its established prognostic significance.

Given that the p53 protein antibody reacts with both the wild-type and mutant-type p53 proteins [33], the statistically significantly more frequent accumulation of p53 protein in the basal parts of the crypts of SSLs without dysplasia (*p* = 0.0063) (Figure 6E) confirms the regulatory role of the p53 protein in activating specific genes and suppressing others, further confirming the neoplastic nature of serrated lesions regardless of the absence of cytological dysplasia. The observation of certain authors that overexpression of nuclear p53 protein in TSA is restricted to areas with conventional dysplasia [34] was not confirmed by our analysis; instead, nuclear p53 protein expression was present along the entire crypt length, including areas exhibiting the classic serrated type of dysplasia (Figure 6F), which was statistically significant compared to other serrated lesions (*p* = 0.0009).

In general, increased p53 expression rises progressively from low-grade dysplasia (45%) to high-grade dysplasia (53%) and subsequent malignant transformation of serrated lesions (61%) [20]. This step-wise accumulation is in accordance with the results of this study, where expression in the upper half of the crypt was present in 11% of sessile serrated lesions without dysplasia and 18% of sessile serrated lesions with dysplasia, while in malignantly transformed serrated lesions, it reached pancryptic positivity in 25% of cases (Figure 6H). It has been reported that tumor localization can influence biological behavior and that the driving mechanisms of carcinogenesis can vary depending on the anatomical localization within the digestive tract [35]. TP53 mutations are more frequently observed in serrated lesions of the distal colon [36], which can be linked to TSAs, which in our study had pancryptic positivity in 50% of cases, while in our previous study, all TSAs had a rectosigmoid localization [2]. Additionally, other studies have reported p53 positivity in TSA in the range of 40–60.5% [19,20].

The differences in percentages present between our study and others are a consequence of certain limitations that are unequivocally present. Our study is retrospective in nature, single-center, and based on archived biopsy and surgical material from a single institution, which inherently limited the sample sizes in several diagnostically critical subgroups, such as traditional serrated adenomas and malignantly transformed lesions. Due to the lack of longitudinal follow-up and long-term clinical outcome data, the evaluated marker panel cannot be utilized for direct prognostic risk prediction regarding malignant transformation; instead, its clinical utility must be interpreted strictly within the bounds of cross-sectional diagnostic associations. Furthermore, because no non-serrated comparator group was included, the diagnostic performance estimates must be interpreted with caution. We also explicitly acknowledge an increased risk of Type I error due to multiple statistical testing across these subgroups. Lastly, p53 immunohistochemistry represents only a phenotypic surrogate for underlying TP53 alterations and was not confirmed via molecular sequencing or mutation analysis. While the histological interpretation and classification of the serrated lesions in our study did not exhibit interobserver variability, unlike some previous reports [12], these cumulative limitations highlight that our findings represent exploratory diagnostic patterns that require cautious interpretation.

## 5. Conclusions

In conclusion, the examined immunohistochemical markers show differential expression patterns across colorectal serrated lesion subtypes and may support histopathological assessment in selected, morphologically challenging cases. However, given the retrospective design, small subgroup sizes, and lack of clinical outcome data, further validation in larger, prospectively collected cohorts with appropriate non-serrated control lesions and longitudinal follow-up is required to definitively establish their broader prognostic or risk-prediction utility.

## Figures and Tables

**Table 1:** Diagnostic performance of immunohistochemical patterns in serrated colorectal lesions.

Marker	Analyzed Pattern	Target Diagnosis	Numerator/Denominator	Sensitivity (95% CI)	Specificity (95% CI)	*p*-Value (Statistical Test)
**MUC2**	Pan-cryptic positivity	Serrated Lineage	103/109	94.5% (88.5–97.5%)	N/A *	N/A *
**Ki-67**	Pan-cryptic positivity	TSA	6/6 (Sens) 96/103 (Spec)	100.0% (61.0–100.0%)	93.2% (86.6–96.7%)	<0.001 (Linear-by-Linear)
**MUC5AC**	Loss of expression	All Advanced Lesions **	24/76 (Sens) 33/33 (Spec)	31.6% (22.4–42.6%)	100.0% (89.6–100.0%)	0.003 (Fisher’s Exact)
**MUC5AC**	Loss of expression	Dysplasia/Malignancy	18/46 (Sens) 63/63 (Spec)	39.1% (26.5–53.5%)	100.0% (94.3–100.0%)	<0.001 (Fisher’s Exact)
**p53**	Pan-cryptic positivity	TSA/Malignancy	5/14 (Sens) 95/95 (Spec)	35.7% (16.3–61.2%)	100.0% (96.1–100.0%)	0.020 (Linear-by-Linear)

Table notes and definitions: Numerator/Denominator: Represents the exact number of true positive cases over the total target cases (for Sensitivity), and true negative cases over the total non-target cases (for Specificity). *p*-values are derived from the respective overall association tests (Fisher’s Exact Test with Monte Carlo correction or Linear-by-Linear Association trend test) as described in the Section 2. 95% CI: 95% Confidence Intervals calculated using the Wilson score method. * N/A (Not Applicable): Specificity for MUC2 could not be established because the study population consisted entirely of serrated lineage lesions, with no non-serrated control group (e.g., conventional tubular adenomas) available as a baseline. ** All Advanced Lesions: Includes SSL with dysplasia, TSA, mixed serrated lesions, and malignantly transformed lesions.

## Data Availability

The original contributions presented in this study are included in the article. Further inquiries can be directed to the corresponding author.

## References

[1] WHO Classification of Tumours Editorial Board (2019). Digestive System Tumours.

[2] Ilić I., Ranđelović P., Mijović Ž., Jovičić Milentijević M., Radovanović Dinić B., Cvetković J. (2023). Significance of Micromorphological Characteristics and Expression of Intermediate Filament Proteins CK7 and CK20 in the Differential Diagnosis of Serrated Lesions of the Colorectum. Gastroenterol. Insights.

[3] Florea M.-A., Becheanu G., Niculae A., Dobre M., Costache M. (2025). Immunohistochemical insights into the pathogenesis of colonic sessile serrated lesions. Arch. Clin. Cases.

[4] Ijspeert J.E.G., Nolthenius C.J.T., Kuipers E.J., E van Leerdam M., Nio C.Y., Thomeer M.G.J., Biermann K., van de Vijver M.J., Dekker E., Stoker J. (2016). CT-colonography vs. colonoscopy for detection of high risk sessile serrated polyps. Am. J. Gastroenterol..

[5] Mezzapesa M., Losurdo G., Celiberto F., Rizzi S., d’Amati A., Piscitelli D., Ierardi E., Di Leo A. (2022). Serrated Colorectal Lesions: An Up-to-Date Review from Histological Pattern to Molecular Pathogenesis. Int. J. Mol. Sci..

[6] Gibson J.A., Hahn H.P., Shahsafaei A., Odze R.D. (2011). MUC expression in hyperplastic and serrated colonic polyps: Lack of specificity of MUC6. Am. J. Surg. Pathol..

[7] Behzatoğlu K. (2026). The Power of MUC2 in Mucinous Adenocarcinomas. Pathobiology.

[8] Liu K., Sachar M., Popov V., Pei Z., Quarta G. (2025). Mucin 5AC as a Biomarker for Sessile Serrated Lesions: Results from a Systematic Review and Meta-Analysis. Clin. Transl. Gastroenterol..

[9] Tadepalli U.S., Feihel D., Miller K.M., Itzkowitz S.H., Freedman J.S., Kornacki S., Cohen L.B., Bamji N.D., Bodian C.A., Aisenberg J. (2011). A morphologic analysis of sessile serrated polyps observed during routine colonoscopy (with video). Gastrointest. Endosc..

[10] Khaidakov M., Lai K.K., Roudachevski D., Sargsyan J., Goyne H.E., Pai R.K., Lamps L.W., Hagedorn C.H. (2016). Gastric Proteins MUC5AC and TFF1 as Potential Diagnostic Markers of Colonic Sessile Serrated Adenomas/Polyps. Am. J. Clin. Pathol..

[11] Spring K.J., Zhao Z.Z., Karamatic R., Walsh M.D., Whitehall V.L., Pike T., Simms L.A., Young J., James M., Montgomery G.W. (2006). High prevalence of sessile serrated adenomas with BRAF mutations: A prospective study of patients undergoing colonoscopy. Gastroenterology.

[12] Kamba E., Murakami T., Tsugawa N., Otsuki Y., Nomura K., Kadomatsu Y., Fukushima H., Saito T., Shibuya T., Yao T. (2025). Endoscopic and Clinicopathological Features of a Colorectal Mucin-Rich Variant of Traditional Serrated Adenoma. Digestion.

[13] Imura T., Miyagawa-Hayashino A., Honda M., Harusato A., Katada K., Konishi E. (2025). Disease-specific alterations of the enteric nervous system in precancerous colonic mucosa and their implications for mucin regulation. Sci. Rep..

[14] Kurosawa T., Murakami T., Yamashiro Y., Terukina H., Hayashi T., Saito T., Nojiri S., Sakamoto K., Nagahara A., Yao T. (2022). Mucin phenotypes and clinicopathological features of colorectal adenocarcinomas: Correlation with colorectal adenocarcinoma with enteroblastic differentiation. Pathol. Res. Pract..

[15] Torlakovic E.E., Gomez J.D., Driman D.K., Parfitt J.R., Wang C., Benerjee T., Snover D.C. (2008). Sessile serrated adenoma (SSA) vs. traditional serrated adenoma (TSA). Am. J. Surg. Pathol..

[16] Pai R.K., Bettington M., Srivastava A., Rosty C. (2019). An update on the morphology and molecular pathology of serrated colorectal polyps and associated carcinomas. Mod. Pathol..

[17] Osakabe M., Yamada N., Sugimoto R., Uesugi N., Nakao E., Honda M., Yanagawa N., Sugai T. (2025). The pattern-based interpretation of p53 immunohistochemical expression as a surrogate marker for TP53 mutations in colorectal cancer. Virchows Arch..

[18] Murakami T., Akazawa Y., Yatagai N., Hiromoto T., Sasahara N., Saito T., Sakamoto N., Nagahara A., Yao T. (2018). Molecular characterization of sessile serrated adenoma/polyps with dysplasia/carcinoma based on immunohistochemistry, next-generation sequencing, and microsatellite instability testing: A case series study. Diagn. Pathol..

[19] Pap Z., Pávai Z., Dénes L., Brînzaniuc K., Jung I. (2011). Hyperplastic polyps and serrated adenomas: Precancerous lesions with mixed immunophenotype. Rom. J. Morphol. Embryol..

[20] Cazacu S.M., Iordache S., Iovănescu V.F., Streba L., Neagoe C.D., Busuioc C.J., Cârţu D., Florescu M.M. (2025). The role of CDX2, MUC5AC, and p53 in the evaluation of the progression of serrated lesions toward colorectal carcinoma. Rom. J. Morphol. Embryol..

[21] Kim K.M., Lee E.J., Ha S., Kang S.Y., Jang K.T., Park C.K., Kim J.Y., Kim Y.H., Chang D.K., Odze R.D. (2011). Molecular features of colorectal hyperplastic polyps and sessile serrated adenoma/polyps from Korea. Am. J. Surg. Pathol..

[22] Jass J.R. (2002). Serrated adenoma of the colorectum. Curr. Diagn. Pathol..

[23] Živković V., Gligorijević J., Djordjević B., Ilić I., Petrović A., Krstić M. (2009). Expression of cytokeratins 7 and 20, and Ki-67 in serrated adenoma of the colorectum. Virchows Arch..

[24] Chlumská A., Boudová L., Zámecník M. (2006). Sessile serrated adenomas of the large bowel. Clinicopathologic and immunohistochemical study including comparison with common hyperplastic polyps and adenomas. Cesk. Patol..

[25] Turner M.A., Cox K.E., Liu S., Neel N., Amirfakhri S., Nishino H., Hosseini M., Alcantara J.A., Abd El-Hafeez A.A., Lwin T.M. (2023). Specific Targeting and Labeling of Colonic Polyps in CPC-APC Mice with Mucin 5AC Fluorescent Antibodies: A Model for Detection of Early Colon Cancer. Curr. Issues Mol. Biol..

[26] Mikhaleva L.M., Vandysheva R.A., Shakhpazyan N.K., Fedorov E.D., Biryukov A.E., Midiber K.Y., Pechnikova V.V. (2019). Comparative assessment of the expression of Muc 2, Muc 5AC, and Muc 6 in serrated neoplasms of the colon. Arkh. Patol..

[27] Liu C., Walker N.I., Leggett B.A., Whitehall V.L., Bettington M.L., Rosty C. (2017). Sessile Serrated adenomas with dysplasia: Morphological patterns and correlations with MLH1 immunohistochemistry. Mod. Pathol..

[28] Krishn S.R., Kaur S., Smith L.M., Johansson S.L., Jain M., Patel A., Gautam S.K., Hollingsworth M.A., Mandel U., Clausen H. (2016). Mucins and associated glycan signatures in colon adenoma-carcinoma sequence: Prospective pathological implication(s) for early diagnosis of colon cancer. Cancer Lett..

[29] Li A., Goto M., Horinouchi M., Tanaka S., Imai K., Kim Y.S., Sato E., Yonezawa S. (2001). Expression of MUC1 and MUC2 mucins and relationship with cell proliferative activity in human colorectal neoplasia. Pathol. Int..

[30] Fujita K., Hirahashi M., Yamamoto H., Matsumoto T., Gushima M., Oda Y., Kishimoto J., Yao T., Iida M., Tsuneyoshi M. (2010). Mucin core protein expression in serrated polyps of the large intestine. Virchows Arch..

[31] Cox K.E., Liu S., Lwin T.M., Hoffman R.M., Batra S.K., Bouvet M. (2023). The Mucin Family of Proteins: Candidates as Potential Biomarkers for Colon Cancer. Cancers.

[32] Bettington M., Walker N., Rosty C., Brown I., Clouston A., Wockner L., Whitehall V., Leggett B. (2014). Critical appraisal of the diagnosis of the sessile serrated adenoma. Am. J. Surg. Pathol..

[33] Smith M.L., Fornace A.J. (1995). Genomic instability and the role of p53 mutations in cancer cells (review). Curr. Opin. Oncol..

[34] Bettington M.L., Walker N.I., Rosty C., Brown I.S., Clouston A.D., McKeone D.M., Pearson S.-A., Klein K., A Leggett B., Whitehall V.L. (2015). A clinicopathological and molecular analysis of 200 traditional serrated adenomas. Mod. Pathol..

[35] Van Meer A., Gilis J., Oosterlinck B., Vandamme T., De Man J., De Winter B.Y., Smet A. (2026). Clinicopathological and prognostic significance of mucin signatures in lower gastrointestinal cancer—A systematic review and meta-analysis. Br. J. Cancer.

[36] Tsugawa N., Kamba E., Murakami T., Otsuki Y., Nomura K., Kadomatsu Y., Fukushima H., Sugimoto K., Saito T., Shibuya T. (2025). Discrete Immunohistochemical and Clinicopathological Features of Serrated Adenocarcinoma between the Proximal and Distal Colon. Digestion.

